# CRISPR/Cas9: a double-edged sword when used to combat HIV infection

**DOI:** 10.1186/s12977-016-0270-0

**Published:** 2016-05-27

**Authors:** Chen Liang, Mark A. Wainberg, Atze T. Das, Ben Berkhout

**Affiliations:** McGill University AIDS Centre, Lady Davis Institute, Jewish General Hospital, Montreal, QC H3T1E2 Canada; Departments of Medicine, Microbiology and Immunology, McGill University, Montreal, QC H3A 2B4 Canada; Laboratory of Experimental Virology, Department of Medical Microbiology, Center for Infection and Immunity Amsterdam (CINIMA), Academic Medical Center, University of Amsterdam, Meibergdreef 15, 1105 AZ Amsterdam, The Netherlands

The major barrier to eradication of HIV infection is the latent viral reservoir that persists despite long-term highly active antiretroviral therapy (HAART). The main reason for the existence of latently infected cells is that proviral DNA becomes integrated into the cellular genome. Theoretically, the elimination of proviral DNA from every infected cell should therefore be able to cure HIV infection. This concept has been tested in studies that employed designed recombinases [1], zinc finger nucleases (ZFNs) and transcription activator-like effector nucleases (TALENs) bearing sequence-specific DNA-binding modules that recognize HIV DNA sequences [2]. In addition, the recent development of the bacterial adaptive immune system CRISPR/Cas9 for editing of genes in mammalian cells [3, 4] quickly led to the use of this new genome editing technology to try to inhibit and eliminate infection by different viruses, including HIV-1 [5].

Cas9 is an endonuclease that cleaves double-stranded DNA in a sequence- specific manner. Cas9 associates with a guide RNA of which the first 20 nucleotides pair with the target DNA. In addition to this single guide RNA (sgRNA), Cas9 also needs to recognize a multi-nucleotide region that is adjacent to the 3′ end of the target DNA, which is termed PAM (protospacer adjacent motif). Several labs have designed sgRNAs to program Cas9 to cleave different regions of HIV-1 DNA that include either essential viral genes or the viral long terminal repeat (LTR). Profound suppression of HIV-1 production and infection was reported in different cell types including latently infected CD4+ T cell lines, primary CD4+ T cells and induced human pluripotent stem cells [6–11].

Despite the promising possibility that CRISPR/Cas9 could be used to inactivate or even delete proviral DNA from HIV-1 infected cells, an important unanswered question is whether and how HIV-1 might escape from the programmed CRISPR/Cas9 attack, a topic that is fundamental to attempts aimed at HIV treatment and prevention, including the use of small molecule-based antiretroviral therapy and HIV vaccines. Two recent publications by Wang G et al. 2016 and Wang Z et al. 2016 have now provided unexpected answers to these questions [12, 13].

Both groups performed HIV-1 evolution experiments in CD4+ T cells that stably expressed both Cas9 and one of several sgRNAs that target different regions of the HIV-1 genome. Although prominent virus inhibition was apparent in transient assays, all infections yielded high levels of HIV-1 production after a variable time. Rapid escape was observed when non-conserved HIV-1 sequences were attacked, but it did take longer for HIV-1 to escape from Cas9/sgRNAs that targeted the more conserved viral DNA sequences [12]. It could be expected that HIV-1 would change the sequence of the viral DNA that is targeted by sgRNA or the PAM sequence, knowing how HIV-1 escapes from a sequence-specific RNA interference attack [14–16]. Indeed, when the targeted viral DNA regions were sequenced, mutations were identified that interfered with sgRNA recognition.

**Fig. 1:**
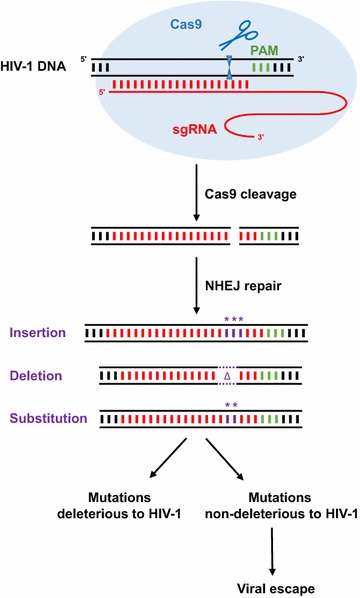
HIV-1 escapes from Cas9/sgRNA. Cas9 is directed to HIV-1 DNA by sgRNA, then cleaves the target DNA at a position 3 nucleotides from the PAM. When the NHEJ machinery repairs the double-stranded DNA break, short nucleotide insertions, deletions and substitutions are generated at the cleavage site. Many of these mutations abrogate viral gene function and thus kill the virus. However, some mutations do not harm the virus and can promote viral escape. PAM, protospacer adjacent motif. sgRNA, single guide RNA. *NHEJ* non-homologous end joining. * substituted or inserted nucleotides. Δ, deleted nucleotides

Then came the unexpected observation. The majority of the resistance mutations appeared to cluster at the site at which Cas9 was designed to cleave the viral DNA, even though the sgRNA binding site is much bigger. Another striking feature was the frequent occurrence of insertions and deletions (indels), at least for the less conserved viral target sequences. This suggests that these mutations are not the result of mistakes by the error-prone viral reverse transcriptase (RT) enzyme but rather represent mutations that are generated by the cellular non-homologous end joining (NHEJ) machinery that repairs broken DNA (Fig. 1). This possibility was confirmed by deep sequencing analysis that showed that a number of the resistance-conferring mutations in the viral escape variants indeed matched the mutations that were introduced into the viral DNA in CD4+ T cells that had been infected by HIV-1 for only 36 h [13]. Therefore, following sgRNA-targeted Cas9 cleavage, the error-prone NHEJ repair machinery generates a variety of mutations at the cleavage site. Some mutations abrogate the function of viral DNA and will not be selected, while others will be selected because they are not deleterious to the virus, yet generate resistance to Cas9/sgRNA attack because the target DNA sequence is changed. For some conserved targets, the indel type of mutations was apparently not compatible with virus replication, and in this case nucleotide substitutions appeared after a longer period that may have been introduced by NHEJ or regular RT mutagenesis.

Knowing how HIV-1 acquires resistance to Cas9/sgRNA may spur the development of strategies that might overcome this unique viral escape mechanism. One solution may be to program Cas9 with multiple sgRNAs that target conserved viral DNA regions. Similar combinatorial strategies were effective for regular antiviral drugs and RNAi-mediated gene therapy to sustainably suppress viral replication [15]. Liao et al. did show that multiplexed targeting of HIV-1 DNA led to much stronger suppression of HIV-1 infection, albeit that possible viral escape was not pursued [8]. Another approach might involve the engineering of new Cas9 variants that are able to cleave DNA outside of the target sequence, so that the mutations arising from NHEJ repair will not prevent Cas9/sgRNA binding and DNA cleavage, thus not leading to viral resistance. Such an approach might be feasible given recent progress at creating Cas9 variants that are able to recognize different PAMs, which demonstrates the flexibility of the system [17–19]. Besides modifying Cas9, the advent of new CRISPR or CRISPR-like enzymes may also provide such a solution. For example, the newly discovered Cpf1 protein acts like Cas9, but in contrast to Cas9 that cleaves the DNA in the “seed” region adjacent to the PAM that is crucial for sgRNA recognition, Cpf1 cleaves in the more distal region of the target sequence that is less critical for sgRNA binding [20, 21]. Yet another anti-escape solution may be the suppression of the NHEJ activity, which can be achieved with anti-cancer drugs that target enzymes of the NHEJ machinery [22]. Solutions to overcome HIV-1 resistance to CRISPR/Cas9 may also advance the application of CRISPR/Cas9 toward suppression of other viral infections.

The findings reported in these two recent studies should not impede the use of alternative CRISPR/Cas9 strategies to combat HIV infection. For example, CRISPR/Cas9 has been proposed for inactivation of the co-receptor genes CXCR4 and CCR5 to render cells refractory to HIV-1 infection [23, 24]. If HIV-1 were able to escape from such a strategy, CRISPR/Cas9 would not directly contribute to the generation of resistance mutations. It is recognized that in vivo disruption of CXCR4, rather than CCR5, may interfere with the immune function of T cells. A second example is activation of latent HIV-1 proviruses by Cas9 mutants that lack nuclease activity and that carry transcription activation domains from transcription activator proteins [25–27]. When guided by sgRNAs to the HIV-1 LTR promoter, these Cas9 variants should be able to stimulate viral gene expression and drive HIV-1 out of latency. The lack of nuclease activity would preclude these Cas9 variants from facilitating viral escape.

In addition to viral escape, several other limitations of the CRISPR/Cas9 system also warrant consideration. First, despite the fact that CRISPR/Cas9 has been shown to be effective in excision of HIV-1 proviral DNA either from acutely or latently infected cells [6–8, 10], none of these studies have tested this ability of CRISPR/Cas9 in the context of resting CD4 T cells from infected individuals. Second, we recognize that single gRNA-mediated suppression of HIV-1 replication is less pronounced compared to current therapies with small molecule inhibitors. It is possible that CRISPR/Cas9 could be combined with HAART to clear latently infected cells, which HAART on its own has been unable to achieve. Unlike antiviral drugs, CRISPR/Cas9 as a gene therapy approach has the potential to durably protect cells against HIV-1. The in vivo delivery of the CRISPR/Cas9-encoding genes might be a challenge, but the lentiviral vector system is efficient at transducing both CD4 T cells and CD34 hematopoietic stem cells. However, the integrating lentiviral vector might pose a risk of insertional oncogenesis. One recent solution to this challenge might be the use of virus-like particles that package Vpr-Cas9/sgRNA for transient delivery of this complex into target cells [28].

It is not readily clear whether some of these limitations of the CRISPR/Cas9 system can be overcome by switching to either the TALEN or ZFN systems. Although both the latter are more simple in that they do not have an RNA component, they are more difficult to program in terms of precise sequence specificity [29]. Importantly, ZFN-mediated cleavage of HIV-1 DNA can also lead to the generation of resistance mutations [30]. Nonetheless, knowledge that has been acquired using ZFN or TALEN to edit viral or cellular genes might facilitate the application of CRISPR/Cas9 in similar settings.

Recognition of the limitations of the CRISPR/Cas9 system will only advance its potential for future widespread application while suggesting strategies for improvement and optimization. The rapid progress in the CRISPR field and the discovery of new genetic tools ensures that this novel genome editing system will continue to provide surprises and excitement while leading to new and novel ways of attaining control of viral infections.
